# Carbonic anhydrase inhibition selectively prevents amyloid β neurovascular mitochondrial toxicity

**DOI:** 10.1111/acel.12787

**Published:** 2018-06-05

**Authors:** María E. Solesio, Pablo M. Peixoto, Ludovic Debure, Stephen M. Madamba, Mony J. de Leon, Thomas Wisniewski, Evgeny V. Pavlov, Silvia Fossati

**Affiliations:** ^1^ Department of Basic Sciences New York University College of Dentistry New York New York; ^2^ Department of Natural Sciences Baruch College Graduate Center The City University of New York New York New York; ^3^ Department of Psychiatry New York University School of Medicine New York New York; ^4^ Department of Neurology Center for Cognitive Neurology New York University School of Medicine New York New York

**Keywords:** Alzheimer's disease, amyloid β, carbonic anhydrase inhibitors, acetazolamide, methazolamide, mitochondria

## Abstract

Mounting evidence suggests that mitochondrial dysfunction plays a causal role in the etiology and progression of Alzheimer's disease (AD). We recently showed that the carbonic anhydrase inhibitor (CAI) methazolamide (MTZ) prevents amyloid β (Aβ)‐mediated onset of apoptosis in the mouse brain. In this study, we used MTZ and, for the first time, the analog CAI acetazolamide (ATZ) in neuronal and cerebral vascular cells challenged with Aβ, to clarify their protective effects and mitochondrial molecular mechanism of action. The CAIs selectively inhibited mitochondrial dysfunction pathways induced by Aβ, without affecting metabolic function. ATZ was effective at concentrations 10 times lower than MTZ. Both MTZ and ATZ prevented mitochondrial membrane depolarization and H_2_O_2_ generation, with no effects on intracellular pH or ATP production. Importantly, the drugs did not primarily affect calcium homeostasis. This work suggests a new role for carbonic anhydrases (CAs) in the Aβ‐induced mitochondrial toxicity associated with AD and cerebral amyloid angiopathy (CAA), and paves the way to AD clinical trials for CAIs, FDA‐approved drugs with a well‐known profile of brain delivery.

## 1 INTRODUCTION

Alzheimer's disease (AD) is the most prevalent type of dementia in the developed world. Despite the enormous efforts made by the scientific community, an effective therapeutic strategy against AD has yet to be developed. The importance of mitochondrial dysfunction in the pathogenesis of AD and other neurodegenerative diseases has been increasingly recognized (Mancuso, Coppede, Murri & Siciliano, 2007; Swerdlow, Burns & Khan, 2010). A causal relationship has been found between mitochondrial dysfunction and amyloid β (Aβ)‐induced neuronal and vascular degeneration (Abramov, Scorziello & Duchen, 2007; Fossati et al., 2010; Swerdlow et al., 2010). Indeed, mitochondrial pathology, oxidative stress, and energy metabolism impairment are implicated in the pathogenesis of AD and present in patients with AD and transgenic animal models, preceding formation of Aβ plaques, cell death, and memory loss (Beal, 2005). Little attention has been paid to the mitochondrial molecular/biochemical pathways leading to AD. The scientific community emphasizes the need to explore mitochondrial pathways to provide solutions to unanswered questions in the prevention and treatment of the disease.

Mitochondrial‐specific therapies are emerging as promising therapeutic tools. It is interesting that mitochondrial therapies have shown beneficial effects in different models of neurodegenerative pathologies, where mitochondrial dysfunction and apoptotic cell death are known to be involved, such as AD (Fossati, Ghiso & Rostagno, 2012b; Moreira, Carvalho, Zhu, Smith & Perry, 2010), Parkinson's disease (Solesio, Prime et al., 2013; Solesio, Saez‐Atienzar, Jordan & Galindo, 2012), and Huntington's disease (Solesio, Saez‐Atienzar, Jordan & Galindo, 2013).

Carbonic anhydrases (CA) are enzymes involved in the reversible conversion of carbon dioxide and water into bicarbonate and protons. They are present in all the vertebrates, showing different intracellular locations and regulating pH and ion transport. CA‐VA and CA‐VB have a mitochondrial localization (Ghandour, Parkkila, Parkkila, Waheed & Sly, 2000). CA‐II, known as cytoplasmic, was also recently shown by proteomic profiling to be increased in brain mitochondria in aging and neurodegeneration (Pollard, Shephard, Freed, Liddell & Chakrabarti, 2016). CA inhibitors (CAIs) are used to treat a variety of disorders including glaucoma, epilepsy, neuropsychiatric disorders, and acute mountain sickness (Aggarwal, Kondeti & McKenna, 2013; Fossati et al., 2016; Huang et al., 2010).

In this study, we examine multiple mitochondrial pathways of amyloid toxicity in neuronal and cerebral endothelial cells (ECs), and evaluate CAIs as active regulators of these processes. We analyze changes in mitochondrial membrane potential, production of ATP, emission of ROS (reactive oxygen species), mitochondrial and cytoplasmic calcium influx, as well as activation of caspase 9 and cell death. While unveiling mechanistic insights into the deleterious mitochondrial actions of amyloid, we propose and test a novel therapeutic approach for preventing these deleterious events. We analyze the role of the CAI methazolamide (MTZ) and, for the first time, its analog acetazolamide (ATZ), on specific Aβ‐mediated pathways of mitochondrial dysfunction and apoptotic cell death, in both neuronal cell lines and microvascular ECs, challenged, respectively, with Aβ42 and the vasculotropic Aβ40‐Q22 (Fossati, Ghiso & Rostagno, 2012a).

Importantly, we include the analysis of mitochondrial toxicity in cerebral endothelial cells. The deposition of amyloid (predominantly Aβ40) around cerebral vessels and microvessels, known as cerebral amyloid angiopathy (CAA), is today recognized as an integral part of the disease. In addition to the well‐known neurodegenerative pathology caused by the parenchymal deposition of Aβ (mainly in its 42 amino acids form), CAA is known to cause vascular damage, micro‐ and macro‐ hemorrhage, apoptosis, and dysfunction of the entire neurovascular unit. These neurovascular effects further exacerbate the pathology and progression of the disease (Revesz et al., 2009; Zlokovic, 2008). Mutations in the Aβ peptide generate variants such as the Aβ40‐Q22 mutant, which are associated with CAA, hemorrhagic stroke, and early‐onset dementia in AD familiar forms, induce aggressive endothelial cell damage, and can represent useful tools to study amyloid‐mediated vascular pathology (Fossati et al., 2010).

MTZ was first selected from a drug library for its ability to inhibit cytochrome C (CytC) release from isolated mitochondria, showing beneficial effects in models of Huntington's disease (Wang et al., 2008) and ischemia‐reperfusion injury (Wang et al., 2009). Albeit pointing to a mitochondrial effect of MTZ, the mechanisms of action were not fully clarified. MTZ prevented cell death and CytC release in cellular and mouse models of Aβ‐induced neurodegeneration (Fossati et al., 2016). This is the first study expanding the analysis to other members of the CAIs family and deeply analyzing the mitochondrial mechanism of action of these drugs. Here, we thoroughly examined the effects of two different FDA‐approved and clinically used members of the CAI family (MTZ and ATZ) on Aβ‐mediated mitochondrial damage, and we tested for the first time if the protective effect induced by MTZ on CytC release, the resulting caspase‐9 activation, and apoptosis, were also exerted by an analog CAI, ATZ. The FDA has approved MTZ and ATZ for use in glaucoma decades ago. CAIs are also currently approved for the prevention of acute mountain sickness and related cerebral edema and as diuretics. Furthermore, ATZ is used in the treatment for idiopathic intracranial hypertension and normal pressure hydrocephalus (Alperin et al., 2014). Their use in these neurological disorders as well as in epilepsy (Aggarwal et al., 2013) confirms the ability of these drugs to reach the brain at effective concentrations. Due to the long‐term use of MTZ and ATZ in chronic conditions, the efficacy and the safety of their systemic administration have been widely assessed (Wright, Brearey & Imray, 2008), making clinical trials for CAIs in AD a concrete possibility. Our novel findings on the mitochondrial effects of MTZ and ATZ against neuronal and vascular amyloid toxicity justify the selection of these drugs as a therapeutic strategy for AD and CAA.

## 2 RESULTS

### 2.1 Aβ treatment elicits mitochondrial membrane depolarization and increases mitochondrial H_2_O_2_ production. CAIs counteract both effects

**Figure 1:**
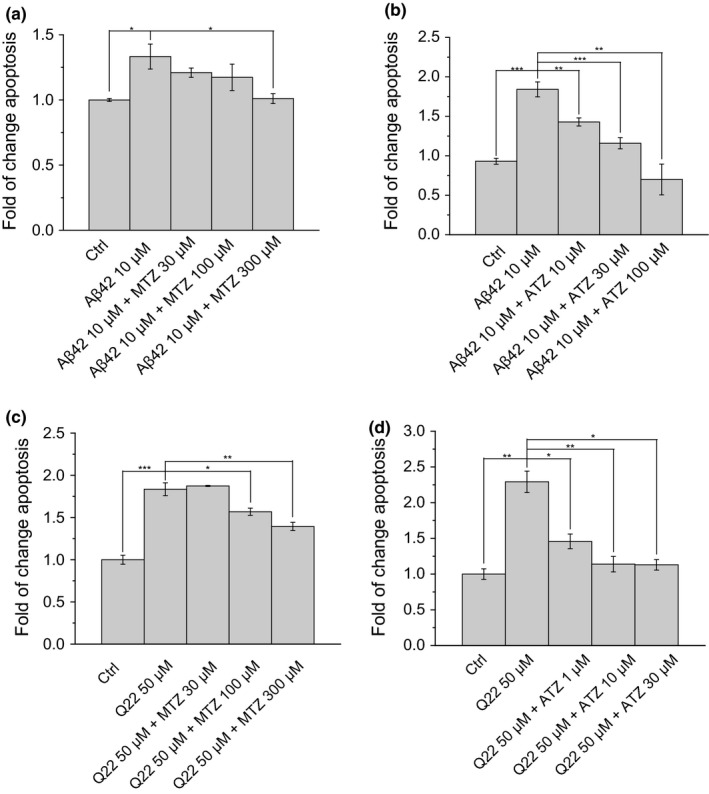
Dose–response curve of Aβ and CAIs. Apoptotic cell death was measured by Cell Death ELISA ^plus^ in neuronal cells (a, b) and microvascular ECs (c, d). Increasing concentrations of CAIs and 10 μM of Aβ42 (a, b) or 50 μM of Aβ40‐Q22 (c, d) were added to the cell cultures. Data in histograms are mean ± *SEM* of at least three independent experiments

**Figure 2:**
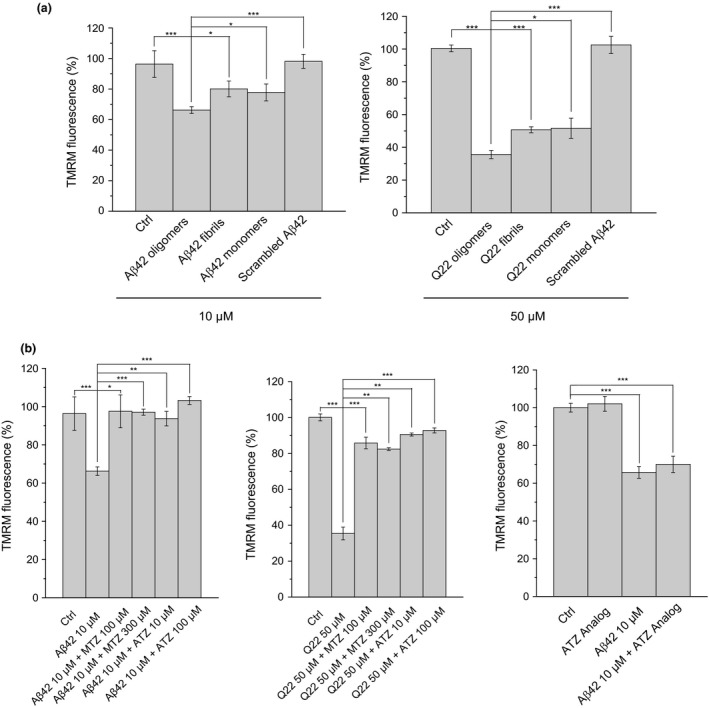
Mitochondrial membrane depolarization induced by Aβ oligomers is prevented by CAIs. Graphs showing mitochondrial membrane potential (ΔΨ) measured by TMRM in SH‐SY5Y neuronal cells (left) and microvascular ECs (right). (a) The monomeric and fibrillar forms of the peptides induced a much lower mitochondrial membrane depolarization. It is interesting that the scrambled form of the peptide did not exert any effect on ΔΨ in any of the cell lines. Aβ42, on its different aggregation states, was always added at 10 μM, while the final concentration of Q22 was 50 μM. (b) ΔΨ in the neuronal cells (left panel) is reduced to about 65% of control cells. The reduction is completely prevented by MTZ and ATZ. In ECs cells (central panel), ΔΨ is reduced to 35% of the control levels by Aβ, and reverted to above 80%, after treatment with the peptide in the presence of CAIs. N‐methyl acetazolamide (100 μM), a structural analog of ATZ unable to inhibit CAs, showed no effect on ΔΨ either under control conditions or in the presence of Aβ, in SH‐SY5Y cells (right panel). Data in histograms are mean ± *SEM* of, at least, three independent experiments

First, to determine the concentrations of MTZ and ATZ effective to decrease the apoptotic effect of the Aβ peptides in both cell lines, we conducted a dose–response experiment measuring DNA fragmentation (Figure 1), which showed that ATZ is about 10‐fold more effective than MTZ at inhibiting apoptosis in both cell types. Afterward, to clarify the molecular mechanisms responsible for the mitochondrial effects of Aβ and to determine whether CAIs exert a protective effect on these processes, we analyzed the main pathways responsible for maintaining mitochondrial function. Preservation of mitochondrial membrane potential (ΔΨ) is an essential element for cell physiology, survival, and energetic function. Indeed, mitochondrial membrane depolarization is known to precede and facilitate apoptotic cell death. We studied the effects of Aβ42 and Aβ40‐Q22 on mitochondrial membrane potential in neuronal cells (SH‐SY5Y) and ECs, respectively. Membrane potential was measured using TMRM fluorescent probe. Our data clearly showed that challenge with aggregated (oligomeric) forms of the Aβ peptides induced a depolarizing effect on the mitochondrial membrane of neuronal cells and ECs, after only 45 min of treatment (Figure 2a). This effect was especially dramatic in the case of microvascular ECs, where ΔΨ was reduced more than 60% by Aβ. It is interesting that the magnitude of the membrane depolarization induced by Aβ fibrils and monomers was significantly lower, compared to the depolarization induced by the oligomeric forms. The scrambled Aβ42 peptide, used as a negative control, did not exert any effect on mitochondrial ΔΨ (Figure 2a). On the other hand, addition of 10 μM of FCCP to completely depolarize mitochondria resulted in loss of fluorescent signal in both cell lines (Supporting Information, Figure S1), confirming that in our experimental conditions, TMRM fluorescence decrease reflects the degree of mitochondrial depolarization.

Both MTZ and ATZ were able to rescue ΔΨ to values similar to those observed in control. 100 μM was used as starting point for MTZ, due to our data indicating an effect of this or higher doses on preventing cell death [(Fossati, Todd, Sotolongo, Ghiso & Rostagno, 2013; Fossati et al., 2016) and Figure 1]. ATZ, used for the first time in this study, was able to prevent the loss of ΔΨ and to maintain the potential at the level of control cells at a significantly lower concentration (10 μM). To demonstrate that this effect was specifically due to the inhibition of CAs by MTZ and ATZ, we used N‐methyl acetazolamide (100 μM), a structural analog of ATZ unable to inhibit CAs. Treatment of SH‐SY5Y cells with the analog exerted no effect on ΔΨ, either under control conditions or in the presence of Aβ.

**Figure 3:**
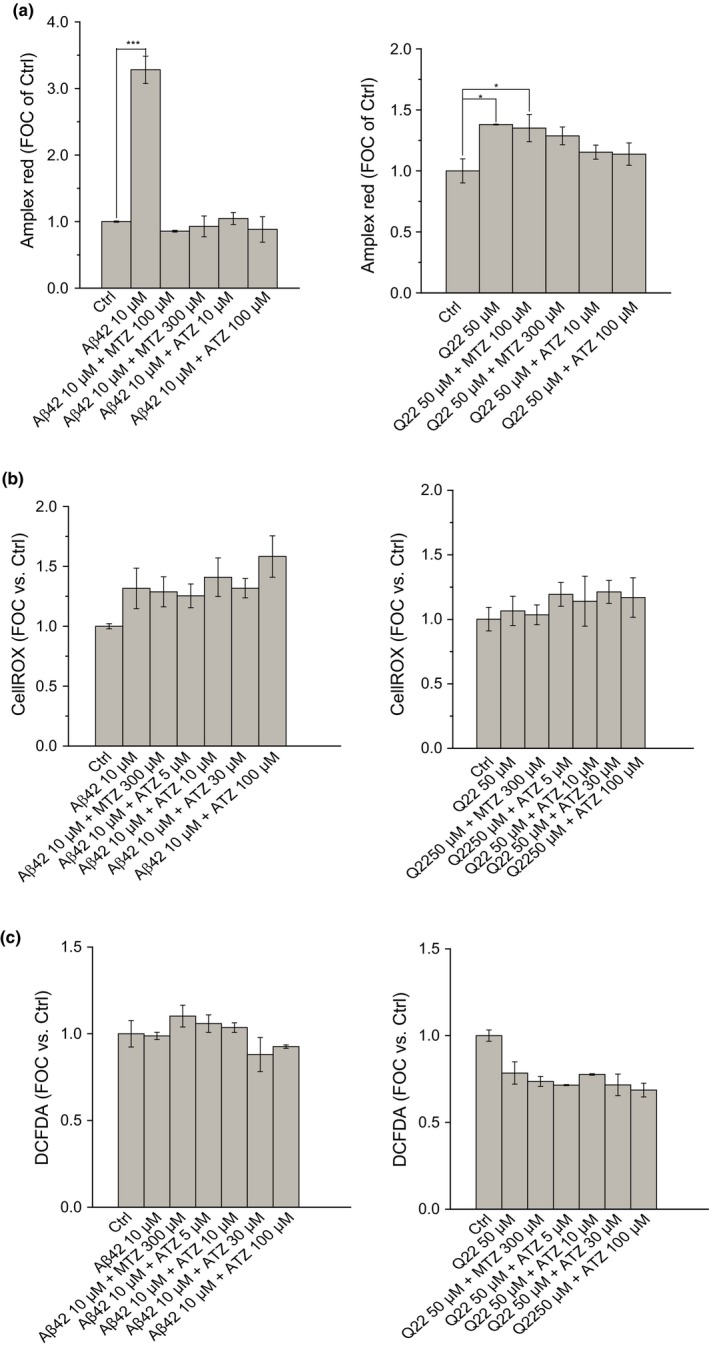
Increase in mitochondrial H_2_O_2_ production in response to Aβ and its inhibition in the presence of CAIs. Effect of Aβ on cellular production. (a) Data show the relative amount of H_2_O_2_ produced by isolated mitochondria expressed as FOC of the control (Ctrl), as measured by Amplex Red in SH‐SY5Y cells (left) and in ECs (right). H_2_O_2_ production significantly increases when cells are treated with Aβ42 10 μM or Q22 50 μM. The release of H_2_O_2_ is prevented when CAIs are added together with Aβ. Both the level of H_2_O_2_ production and the efficiency of its inhibition are more extreme in neuronal cells, compared to ECs. General oxidative stress within the cell, measured by CellROX (b) and DCFDA (c) reagents in neuronal cells (left) and ECs (right), is not significantly affected by Aβ challenge. The addition of CAIs does not significantly change the amount of intracellular ROS present in any of the two cell types. Data in histograms are mean ± *SEM* of at least three independent experiments

Increased production of mitochondrial H_2_O_2_ is a classical signal of mitochondrial dysfunction and an essential mediator of cell death (Singh, Sharma & Singh, 2007). H_2_O_2_ is a membrane permeable second messenger, as well as a potent precursor of other ROS generation (Turrens, 2003). H_2_O_2_ production is also tightly regulated by ΔΨ. We measured the levels of H_2_O_2_ produced by isolated mitochondria purified after neuronal and ECs treatment with Aβ, in the presence or absence of the CAIs. Aβ induced a significant increase in the amount of H_2_O_2_ generated by isolated mitochondria (threefold increase in neuronal cells and about 1.5‐fold increase in ECs), as estimated from Amplex Red fluorescence (Figure 3a). Emission of H_2_O_2_ was inhibited in the presence of ATZ or MTZ. While in neuronal cells both drugs completely reverted the effect of Aβ, ATZ, albeit used at lower concentrations than MTZ, had a more significant effect on ECs (Figure 3a).

### 2.2 Modulation of mitochondrial and cytoplasmic calcium levels

**Figure 4:**
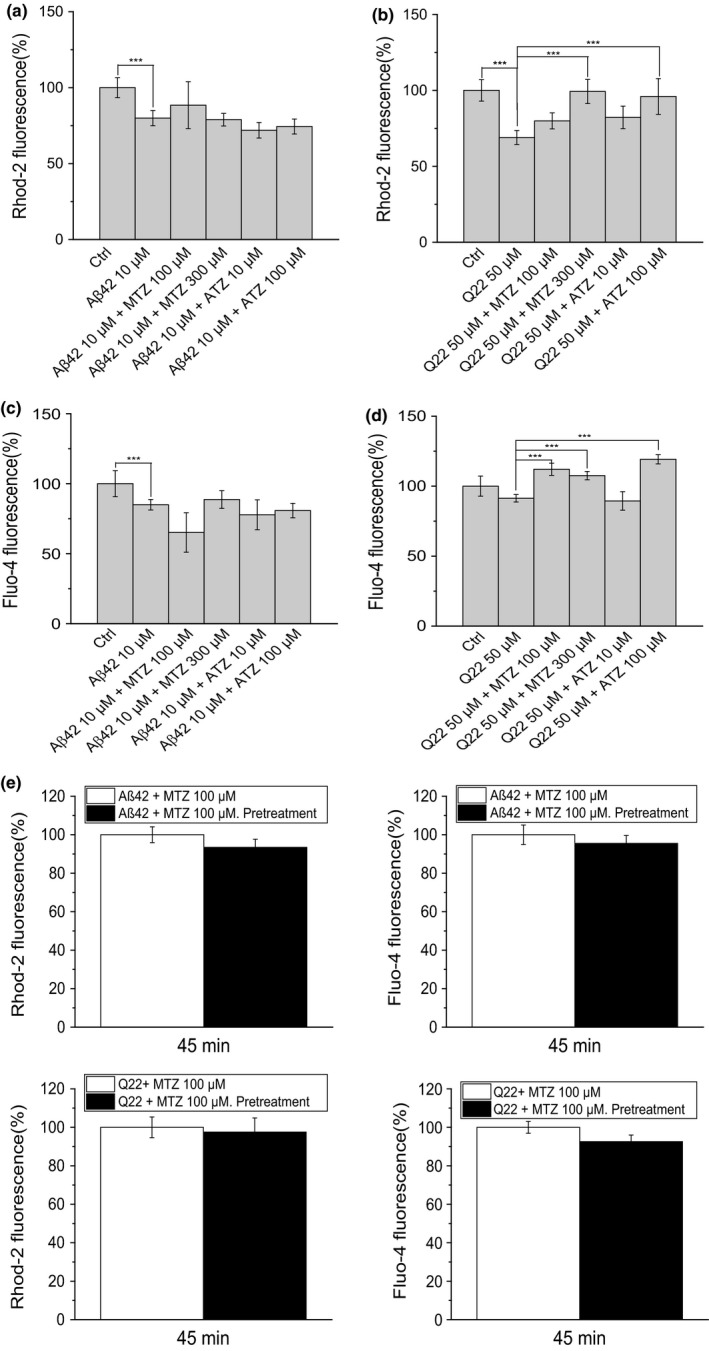
Effect of Aβ on calcium homeostasis and differential impact of CAIs. (a, b) Graphs show mitochondrial calcium accumulated in the presence of Aβ (respectively, Aβ42 10 μM or Q22 50 μM) with or without MTZ (100 or 300 μM) or ATZ (10 or 100 μM) in (a) SH‐SY5Y cells and in (b) ECs. (c, d) Cytoplasmic calcium concentration in the same cell types and under the same treatments is represented. (e) Pretreatment of the cells with the drugs before challenge with Aβ does not significantly affect mitochondrial and cytoplasmic calcium concentration. Data in histograms in (a, b, c, d, and e) are mean ± *SEM* of at least three independent experiments

Fluctuations in mitochondrial‐free Ca^2+^ are usually linked to mitochondrial dysfunction and cell death and they have been previously described in specific cell types challenged with amyloid peptides. Using specialized fluorescent dyes (Rhod‐2/mitochondrial Ca^2+^ and Fluo‐4/cytoplasmic Ca^2+^), we measured the levels of mitochondrial and cytoplasmic‐free Ca^2+^ (Figure 4). Due to their charge, rhodamine‐based calcium probes are known to be preferentially localized in mitochondria (Smithen et al., 2013). This fact was confirmed under our experimental conditions, as shown in Supporting Information, Figure S2a, insert.

Surprisingly, we found that an acute treatment (45 min) with pre‐aggregated oligomeric Aβ peptides decreased mitochondrial‐free calcium levels in both cell types (Figure 4a–b). It is interesting that when we analyzed cytoplasmic Ca^2+^, we detected a similar effect to that observed in the mitochondria, although the decrease was not significant in ECs (Figure 4c–d). CAIs, given together with the peptide for 45 min, did not affect the decreased levels of mitochondrial and cytoplasmic Ca^2+^ in neuronal cells, while in ECs, the highest doses of MTZ and ATZ were able to counteract the effect exerted by the peptide (Figure 4b, d).

To exclude experimental artifacts, we subjected the cells to different loading and washing times, which consistently resulted in decreased levels of free calcium in both mitochondrial and cytoplasmic compartments after Aβ challenge. Pretreatment with the drugs for 3 hr did not produce any significant difference in Ca^2+^ influx, compared to simultaneous addition of peptides and CAIs (Figure 4e). Rhod‐2 has some limitations, mainly related to the fact that it responds to fluctuations in Ca^2+^ concentration not by changing the emission and/or the excitation spectrums, but by variations in the intensity of the fluorescence. In addition, uneven distribution of the dye within mitochondria could also occur when using this probe. However, rhodamine‐based fluorescence probes have been extensively used in the literature as a method to assay mitochondrial calcium (Abramov & Duchen, 2003; Babcock, Herrington, Goodwin, Park & Hille, 1997; Boitier, Rea & Duchen, 1999), and we conducted all the experiments accurately and using the appropriate controls.

### 2.3 Carbonic anhydrase inhibition does not affect intracellular pH or ATP generation

**Figure 5:**
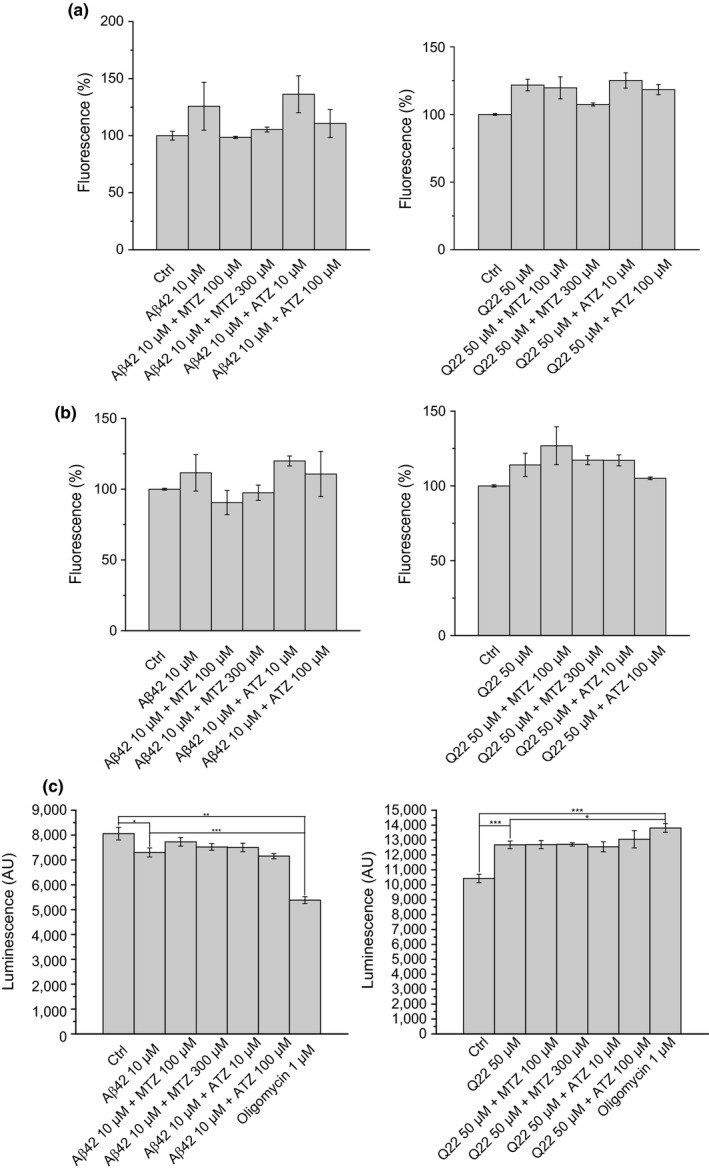
Intracellular pH and ATP production are not affected by CAIs. Measurement of the intracellular pH after 3 hr of treatment with the pre‐aggregated peptide is shown in (a) for SH‐SY5Y and for ECs. The same pH measurement after 16 hr of treatment with Aβ without pre‐aggregation is represented in (b) (Aβ42 10 μM or Q22 50 μM). Cellular pH is not significantly affected by Aβ and CAIs (MTZ 100 or 300 μM and ATZ 10 or 100 μM). (c) Bar histograms showing ATP production in response to Aβ peptides or to peptides in the presence of CAIs for SH‐SY5Y and ECs cell cultures. ATP production is measured by a luminometric assay (CellGlo, Promega) and is represented as A.U. Data in histograms are mean ± *SEM* of, at least, three independent experiments

CA catalyzes the interconversion of CO_2_ and H_2_O to ${\text{HCO}}_{3}^{-}$ and protons, through a reversible reaction (Meldrum & Roughton, 1933). For this reason, upon enzyme inhibition, the intracellular levels of bicarbonate may vary. Because bicarbonate is one of the most important components of the pH buffering system of the human body, changes in its concentration may produce dramatic variations in the intracellular pH. In our conditions, no changes in intracellular pH were elicited by either the Aβ peptides or the CAIs for the duration of the experiments, even if Aβ was aggregated prior to treatments (Figure 5a and b), showing that cellular pH changes do not mediate the CAIs protective effects.

To examine a possible effect of CAIs on proton flux across the inner mitochondrial membrane and energy production, we measured cellular ATP levels in permeabilized cells, using a luciferin‐luciferase assay. Dissimilar results were obtained in SH‐SY5Y and ECs. Treatment of SH‐SY5Y cells with Aβ induced a modest decrease in ATP levels (*p* = 0.05, Figure 5c). These levels remained unchanged upon treatment with MTZ or ATZ in combination with the peptide. As a control, treatment with ATP synthase inhibitor oligomycin induced a sharp decrease (*p* < 0.0001). In contrast, treatment of ECs with the Q22 peptide induced a 21.6% increase in steady‐state ATP levels (*p* = 0.001, Figure 5c). This increase was unaffected by cotreatment with either MTZ or ATZ. It is interesting that oligomycin, similar to Aβ, also increased ATP luminescence in the ECs (*p* = 0.0002).

### 2.4 Aβ‐induced apoptosis and caspase activation are prevented by CAIs

Apoptotic cell death is a well‐known contributor to neurovascular degeneration in AD. CytC release from dysfunctional mitochondria and the resulting caspase‐9 activation are known to play key roles in the apoptotic process. We have recently reported a protective effect of MTZ against apoptotic cell death in models of Aβ‐induced toxicity (Fossati et al., 2016). Here, we tested for the first time if the protective effect induced by MTZ on caspase activation and CytC release was also exerted by the analog CAI ATZ.

**Figure 6:**
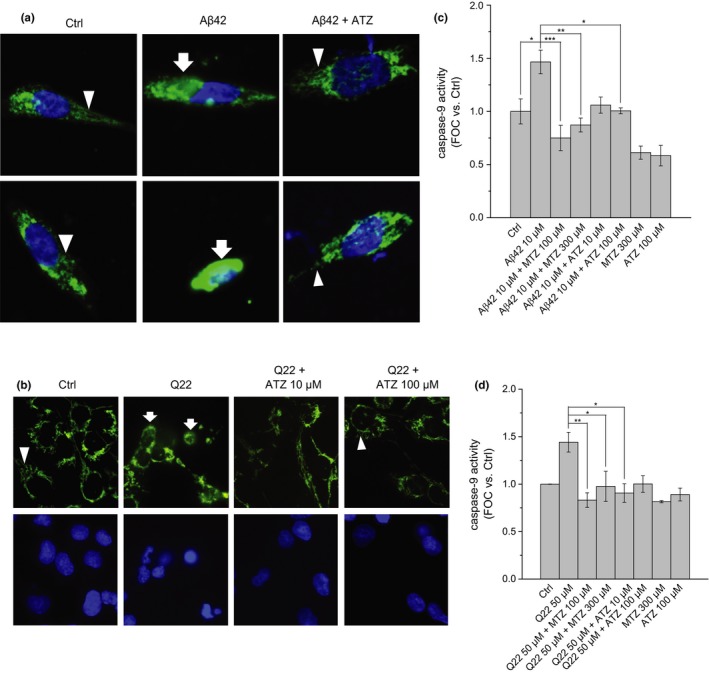
Protective effect of CAIs on Aβ‐induced CytC release and caspase‐9 activation. ATZ prevents CytC release in response to Aβ in (a) SH‐SY5Y and (b) ECs cells. Images were acquired by confocal microscopy after immunocytochemistry. Chain‐like mitochondrial Cyt C (arrowheads) in healthy cells or diffused cytoplasmic CytC (arrows) in cells undergoing apoptosis is stained in green. Nuclei are marked in blue with Hoechst 33342. Nuclear condensation indicates apoptosis. The protective effect of MTZ and ATZ on caspase‐9 activation is shown in (c) for SH‐SY5Y and (d) for ECs cell cultures. Data in histograms are mean ± *SEM* of at least three independent experiments

We analyzed the effect of MTZ on amyloid‐mediated mitochondrial cell death pathways, showing that Aβ‐induced CytC release (Figure 6a and b), caspase‐9 activation (Figure 6c and d), and DNA fragmentation (Figure 1a–d) were inhibited by MTZ starting at 100 μM concentration, which confirmed our recent work (Fossati et al., 2016). Importantly, ATZ was effective in preventing CytC release and caspase‐9 activation, as well as apoptosis, at concentrations 10 times lower than MTZ, starting at concentrations ≤10 μM (Figures 1a–d and 6). ATZ completely reverted Aβ‐induced caspase‐9 activation (Figure 6).

## 3 DISCUSSION

Mitochondrial dysfunction is an early and causal step in AD pathology, tightly linked to neurodegeneration and promoting cognitive impairment (Hirai et al., 2001; Swerdlow, Burns & Khan, 2014; Swerdlow & Khan, 2009; Swerdlow et al., 2010). The apoptotic outcome has been attributed to the pathological effects of Aβ intermediate aggregation species (particularly oligomers and protofibrils) on mitochondrial pathways (Fossati et al., 2010, 2012b). However, the specific biochemical pathways leading to mitochondrial dysfunction in the presence of amyloid, as well as the resulting activation of cell death pathways in neurovascular cells, are still unclear.

Here, we revealed that CAs might be previously unrecognized key targets in these processes. Our results clearly showed induction of mitochondrial membrane depolarization and increased mitochondrial H_2_O_2_ production, in response to Aβ‐challenge, in both neuronal and cerebral microvascular ECs. MTZ and ATZ, two different members of the CAI family, were effective at inhibiting the mitochondrial dysfunction pathways induced by Aβ. Intriguingly, other mitochondrial parameters, such as ATP production and pH, were not equally affected. Moreover, mitochondrial and cytoplasmic calcium flux did not seem to be essential for the mechanism of action of the CAIs.

In the presence of Aβ, mitochondrial membranes were strongly depolarized and mitochondrial production of H_2_O_2_ was increased. These data are concordant with previous reports, showing that the Aβ peptide affects the production of different types of ROS, including H_2_O_2_ (Kaminsky & Kosenko, 2008). The essential role played by mitochondrial H_2_O_2_ production in the activation of the apoptotic pathway in our study is concordant with previous work showing that increments in the generation of this molecule appear early after Aβ‐challenge (Milton, 2004; Tabner et al., 2005). In our model, H_2_O_2_ production and mitochondrial membrane depolarization, which is also a key process in AD pathogenesis (Moreira et al., 2010), appear as primary inductors of Aβ‐mediated apoptotic cell death and as the main targets of CAIs. Both parameters were clearly reverted when CA was inhibited by MTZ and ATZ.

It is interesting that despite the increase in H_2_O_2_ produced by mitochondria isolated after cell treatment with Aβ (Figure 3a), other intracellular ROS measured in whole cells were not increased in response to Aβ in our model (Figure 3b and c). The differences between the effects exerted by the Aβ peptides on H_2_O_2_ release by isolated mitochondria and on other types of ROS measured in whole cells can be explained by the ability of H_2_O_2_ to rapidly cross membranes and be released extracellularly. In line with this hypothesis, while the use of Amplex Red allows to study the amount of H_2_O_2_ released into solution by mitochondria after cell lysis, CellROX and DCFDA only quantify the amount of ROS in the intracellular space, where likely H_2_O_2_ is not continually present, due to its ability to cross membranes. The fact that H_2_O_2_ is highly unstable and that it reacts with lipids and proteins, inducing peroxidation, is also in line with the proposed hypothesis.

To determine whether CAIs affect energy production or proton availability after Aβ challenge, we measured ATP levels in the presence or absence of CAIs. It is interesting that despite cell type differences likely due to different coupling properties, MTZ or ATZ treatment did not affect the steady state of ATP, either in control conditions or in cells treated with Aβ. These results suggest that energy production and proton flux are not involved in the CAIs’ mechanism of action, which seems primarily driven by the prevention of mitochondrial ΔΨ changes and by the reduction in mitochondrial H_2_O_2_ release. Thus, the protective effects of CAIs against amyloid peptides are not due to an increase in mitochondrial respiration. It is interesting that the amyloid peptides alone exerted differential effects on SH‐SY5Y and ECs, inducing a slight decrease in ATP levels in neuronal cells and a substantial increase in cerebral ECs. A possible explanation is that these cell types handle mitochondrial dysfunction by differentially resorting to aerobic glycolysis (Newington et al., 2011). In fact, we can presume fundamentally different glycolytic metabolism for neuronal cells (such as SHSY‐5Y) and ECs. This may explain the different effects induced by the ATP synthase inhibitor oligomycin in our experiments and strengthens our conclusion that the protective mechanisms of CAIs are independent of ATP production.

Maintaining the proper mitochondrial calcium concentration is imperative for cell survival. In fact, mitochondria, jointly with the ER, are the two main organelles in charge of keeping calcium cell homeostasis. However, while in the ER the levels of free calcium are kept within the physiological range by a group of proteins called calsequestrins (MacLennan & Wong, 1971), mitochondria lack any specific protein to exert this action, and the mechanism governing this process is still unclear.

Previous work showed that Ca^2+^ homeostasis is dysregulated in cellular models of AD, as well as in human AD brains (Berridge, Bootman & Lipp, 1998; Celsi et al., 2009; Garwood et al., 2013). Surprisingly, our data showed decreased levels of free calcium, both in mitochondria and in cytoplasm, after the addition of the Aβ peptides. A previous study showed decreased levels of mitochondrial Ca^2+^ under pathological conditions (Granatiero et al., 2015). Discordant results have been reported regarding the effects of Aβ on cellular and mitochondrial calcium, with studies showing data both consistent and contradictory with our findings (Abramov, Canevari & Duchen, 2003, 2004).

The observed effect on mitochondrial and cytoplasmic calcium concentration was partially rescued by the highest concentrations of MTZ and ATZ in microvascular ECs, reaching values similar to those found under control conditions, while no modulation by CAIs was observed in neuronal cells. This is key evidence that mitochondrial CAIs effects on Aβ‐induced toxicity are independent of calcium uptake in both mitochondria and cytoplasm, as even in conditions in which CAIs do not affect mitochondrial and cytoplasmic Ca^2+^ flux (neuronal cells), CAIs are able to inhibit loss of ΔΨ and production of H_2_O_2_, as well as caspase activation and cell death. In the absence of an obvious effect of CAIs on mitochondrial Ca^2+^ levels, we concluded that the observed effects are independent of Ca^2+^ homeostasis. Thus, rather than examining pathways in which fluctuating levels of mitochondrial Ca^2+^ has been shown, as the opening of the mitochondrial permeability transition pore or the ER‐mitochondria interactions, we opted to focus our studies on other mitochondrial parameters affected by CAIs. One of the main consequences of the over dosage of CAIs in humans is the imbalance in the serum electrolyte levels and the resultant change in blood pH (Crandall, Bidani & Forster, 1977). Small increases in the pH of the solutions where MTZ and ATZ are dissolved are also linked to increases in their solubility (Jiang et al., 2013) and may induce changes in the bioavailable concentration of the CAIs, introducing more complexity in our study. Therefore, we monitored possible changes in the intracellular pH upon cell treatment with the CAIs, which could affect Aβ‐induced toxicity. No significant changes in the pH were found (Figure 5b and c). This allowed us to exclude that the protective effects of CAIs were secondary to pH modulation.

As expected, CytC release and caspase‐9 activation were induced by Aβ challenge in both neuronal and microvascular ECs. DNA fragmentation, indicating apoptotic cell death, was also increased by Aβ in both cell types. This is the first study showing that two different CAIs were able to counteract the detrimental effects of Aβ on mitochondrial dysfunction and apoptotic cell death and to analyze their mitochondrial mechanisms of action. While previous findings in neurodegenerative diseases have proposed a role of MTZ in the prevention of mitochondrial dysfunction and CytC release (Fossati et al., 2013, 2016; Wang et al., 2008, 2009), these studies did not explore the potential protective effects of other CAIs, and did not show that the effects were specifically due to CA inhibition. We hypothesized that the prevention of Aβ‐mediated mitochondrial dysfunction may be due to a direct effect on mitochondrial and/or cellular CAs, as shown by the lack of effect of an inactive ATZ analog. Albeit none of the CAIs available today are fully selective for one of the enzymes, both MTZ and ATZ have high activity on mitochondrial CA (CA‐VA and ‐VB) (Supuran, 2008).

In our models, CAIs prevent Aβ‐induced apoptosis by inhibiting loss of ΔΨ and production of H_2_O_2_, as well as CytC release from the mitochondria. A possible mechanism responsible for the prevention of mitochondrial depolarization and H_2_O_2_ production is that pharmacological inhibition of mitochondrial CAs slows down the production of ${\text{HCO}}_{3}^{-}$, limiting Krebs cycle and electron transport chain, and thus reducing the production of H_2_O_2_ and subsequent oxidative stress. This mechanism is also proposed by Shah's group, who showed that inhibition of CAs rescued high‐glucose induced mitochondrial dysfunction, ROS production, and pericyte loss in diabetic mice (Price, Eranki, Banks, Ercal & Shah, 2012; Shah, Morofuji, Banks & Price, 2013). The known effects of CAIs on specific ion channels, aquaporins (Kamegawa, Hiroaki, Tani & Fujiyoshi, 2016), or other receptors which interact with Aβ on mitochondrial or cell membranes may also be mechanisms responsible for the amelioration of Aβ‐induced mitochondrial dysfunction (Aggarwal et al., 2013). More studies will be needed to further clarify these molecular mechanisms.

Our results suggest a new and critical role for CA inhibition in the regulation of Aβ‐induced neuronal and microvascular toxicity, both essential underlying processes of AD etiopathology, through an effect of the CAIs on specific pathways of mitochondrial dysfunction. Importantly, the mitochondrial effects are exerted not only by MTZ, but also by another member of the CAIs family, ATZ, which is effective at even lower concentrations. These concentrations are in the range of those achieved clinically in the brain. Although clinical trials will be required to ultimately demonstrate effects in AD or other dementias, the translatability of our findings will be increased if the protective effects of CAIs are demonstrated in transgenic animal models of amyloidosis. We have previously shown reduced caspase activation and neuronal death after Aβ intracerebral injection in mice treated with a concentration of MTZ of 10 mg/kg, that after allometric scaling is significantly under the maximum recommended dose for human adults (Fossati et al., 2016). Promising studies in transgenic mouse models of amyloidosis are currently ongoing in our laboratory. The physiological relevance of this approach is further highlighted by recent proteomic studies showing increased CAII in the mitochondria in aging and neurodegeneration (Pollard et al., 2016), as well as by our group's recent findings demonstrating the presence of multiple CA enzymes in amyloid plaques within the AD human brain (Drummond et al., 2017).

This study, clarifying for the first time the mitochondrial molecular mechanisms of MTZ and ATZ protection against Aβ toxicity, paves the way for future clinical trials aimed to repurpose these and other FDA‐approved CAIs against AD and dementia.

## 4 EXPERIMENTAL PROCEDURES

### 4.1 Reagents

Dulbecco's modified Eagle medium:F12 1:1 (DMEM:F12), penicillin‐streptomycin, and fetal bovine serum (FBS) were purchased from Gibco‐Invitrogen (Carlsbad, California); anti‐active caspase‐3 antibody from Santa Cruz Biotechnology (Dallas, Texas); protease inhibitors and cell death detection ELISA plus kit from Roche (Basel, Switzerland); endothelial cell growth medium (EBM‐2) and growth supplements from Lonza (Basel, Switzerland); MTZ, ATZ, 1,1,1,3,3,3‐hexafluoro‐2‐propanol (HFIP), dimethyl sulfoxide (DMSO), Triton X‐100 solution, bovine serum albumin (BSA), sucrose, mannitol and Tris‐HCl, phenylmethylsulfonyl fluoride (PMSF), oligomycin, and p‐trifluoromethoxyphenylhydrazone (FCCP) from Sigma‐Aldrich (St. Louis, Missouri); caspase‐Glo 9 assay and Cell Titer‐Glo assay from Promega (Madison, Wisconsin); Amplex Red hydrogen peroxide/peroxidase assay kit and CM‐H2DCFDA (general oxidative stress indicator) from Thermo Fisher (Waltham, Massachusetts); paraformaldehyde 20% (PFA) from Electron Microscopy Sciences, (Hatfield, Pennsylvania); Rhod‐2 AM, Fluo‐4 AM, tetramethylrhodamine methyl ester (TMRM), Hank's balanced salt solution (HBSS), MitoTracker Red, CellROX deep red reagent, pHrodo green AM and Hoechst 333258 from Thermo Fisher (Waltham, Massachusetts); and Alexa Fluor 488 antibody from Abcam (Cambridge, UK).

### 4.2 Cell cultures

The neuroblastoma SH‐SY5Y cell line was purchased from the American Type Culture Collection (ATCC, Manassas, Virginia) and grown as using DMEM:F12 medium supplemented with 20 units/ml penicillin‐streptomycin and 15% (v/v) FBS. Immortalized human brain microvascular endothelial hCMEC/D3 cells (ECs) were obtained from Babette Weksler and grown in complete EBM‐2 medium, containing all the growth supplements (Hydrocortisone, hFGF‐B, VEGF, R3‐ IGF‐1, ascorbic acid, hEGF, GA‐1000, and heparin), 20 units/mL penicillin‐streptomycin, and 5% FBS. Both cell lines were grown in a humidified cell culture incubator, under a 5% CO_2_ atmosphere and at 37°C.

### 4.3 Drug preparation

MTZ and ATZ were both dissolved in DMSO to stock solutions of 300 mM and kept at −20°C until the day of the experiment. The CAIs were thawed at room temperature and then dissolved in the specific medium used in each experiment to the final concentrations, (30 to 300 μM in the case of MTZ and 1 to 100 μM for ATZ).

### 4.4 Aβ peptides

Synthetic Aβ42 was synthesized using N‐tert‐butyloxycarbonyl chemistry by James I. Elliott at Yale University and purified by reverse‐phase high‐performance liquid chromatography on a Vydac C4 column (Western Analytical, Murrieta, California, US). Aβ40‐Q22 (the Dutch genetic variant, containing the E22Q substitution) was synthetized by Peptide 2.0 Inc. (Chantilly, Virginia, USA) and purified by HPLC/MS. Purity was >95%. Scrambled Aβ42 was purchased from Bachem (Torrance, California, USA). Aβ homologs were dissolved to 1 mM in HFIP, incubated overnight to break down preexisting β‐sheet structures (Fossati et al., 2010), and lyophilized. Peptides were subsequently dissolved in DMSO to a 10 mM concentration, followed by the addition of deionized water to 1 mM concentration and by further dilution into the medium in which the experiments were run, to a final concentration of 10 μM in the case of Aβ42 and to 50 μM in the case of the Aβ40‐Q22. Peptide treatments were performed in EBM‐2 supplemented with FBS 1% and in DMEM:F12 with no FBS or 1% FBS, for ECs and SH‐SY5Y cells, respectively.

### 4.5 Peptide preparation

In brief, HFIP‐pretreated and lyophilized peptides were resuspended in DMSO to a 10 mM concentration, immediately prior to use. After that, peptides were directly used in the case of the monomeric preparations and added to the cells at the specified concentrations. Peptide pre‐aggregation to obtain oligomeric and fibrillar preparations was performed following the protocol published by Dahlgren et al. (2002). Aggregates were then added to the cells at the desired concentrations, alone or in cotreatment with the different CAIs. Monomeric, oligomeric, or fibrillary state of the preparations was tested by EM as we previously published (Fossati et al., 2010).

### 4.6 Caspase‐9 activation

Cells were plated at a confluence of 10,000 cells per well in 96‐well luminescence microtiter microplates (Thermo Fisher, Waltham, Massachusetts, US). The day after, cells were treated with the peptides and/or CAIs for the experimental times. After that, caspase‐Glo 9 assay (Promega, Madison, Wisconsin, USA) was performed, following the protocol provided from the manufacturer. Luminescence was measured using a FlexStation 3 Multi‐Mode Microplate Reader (Molecular Devices, Sunnyvale California, USA).

### 4.7 Amplex Red

Cells were plated on 6‐well plates and treated with the peptides and/or CAIs for the experimental times. Plates were centrifuged for 10 min at 200 RCF and washed twice with PBS 1×. Afterward, to isolate the mitochondria, 500 μl of homogenization buffer (75 mM sucrose, 225 mM mannitol, 5 mM Tris‐HCl pH = 7.4, 1 mM PMSF, and protease inhibitor cocktail) was added. Cells were scraped in this buffer, collected in glass tubes, and grinded exactly 80 times with a pellet pestle, keeping everything always on ice. Cells were then centrifuged at 800 RCF for 5 min at 4°C. The supernatant was collected and centrifuged again at 800 RCF for 5 min at 4°C. The supernatant was again collected and centrifuged at 11700 RCF for another 5 min at 4°C. After this, the supernatant was discarded, and the pellet was resuspended in 100 μl of homogenization buffer. The samples were centrifuged again at 11700 RCF for 10 min at 4°C, the supernatant was discarded, and the pellets (mitochondrial fractions) were resuspended on 50 μl of homogenization buffer. A protein concentration assay on mitochondrial protein was performed using the Pierce BCA Protein Assay Kit (Thermo Fisher, Waltham, Massachusetts, USA) and following the instructions provided by the manufacturer. After that, mitochondrial fractions containing equal amounts of mitochondrial proteins were placed on 96‐well absorbance microtiter microplates (Thermo Fisher, Waltham, Massachusetts, USA), and the Amplex Red assay was run, following the instructions provided by the manufacturer. Absorbance was measured by using a FlexStation (Molecular Devices, Sunnyvale California, USA).

### 4.8 Immunocytochemical evaluation of Cytochrome C release

SH‐SY5Y and microvascular ECs cells were plated on 15‐mm optical borosilicate poly‐L‐lysine‐coated sterile glass covers (Thermo Fisher, Waltham, Massachusetts, USA) at a 70% confluence. After 24 hr, cells were treated with the peptides in the presence or absence of MTZ or ATZ for 16 hr. Cells were then washed with PBS, fixed with 4% paraformaldehyde (10 min, RT), washed again, and blocked with 20 mg/ml BSA in PBS containing 0.3% Triton X‐100 (PBST). Slides were further incubated with mouse monoclonal anti‐CytC antibody (BD Biosciences; 1:200 in PBST containing 5 mg/ml BSA; 2 hr, RT) followed by Alexa Fluor 488‐conjugated anti‐mouse IgG (Thermo Fisher, Waltham, Massachusetts, USA) 1:200 in PBST with 5 mg/ml BSA for 1 hr at RT, as previously described fluorescence signals were visualized in a Zeiss LSM 510 laser scanning confocal/Confocor2 microscope using a 40× DIC oil immersion objective and LSM 510 software; acquired images were processed and analyzed using ImageJ (National Institute of Health).

### 4.9 Mitochondrial and cytoplasmic calcium assay

This assay was performed as previously published in F. In brief, cells were plated on 25‐mm optical borosilicate poly‐L‐lysine‐coated sterile glass covers, (Thermo Fisher, Waltham, Massachusetts, USA) at a 70% confluence. 24 hr later, cells were loaded with 5 μM Rhod‐2 AM or 2.5 μM Fluo‐4 AM on HBSS for 45 min. Then, cells were washed twice with HBSS and incubated for an additional 15 min on fresh HBSS, without Rhod‐2 AM or Fluo‐4 AM. After that, HBSS was replaced again by fresh HBSS, glasses were mounted on microscopy chambers, and experiments were conducted, after adding the pre‐aggregated peptides and/or the CAIs. Cells were mounted on Sykes‐Moore chambers (BellCo, Vineland, New Jersey, USA) and imaged every 15 s for 45 min, at a 20× magnification, using a Nikon fluorescent microscope (Chiyoda, Tokyo, Japan). The calcium ionophore ionomycin (5 μM) was added at the end of the experiments to test whether the cells were still functional and able to maintain calcium gradient between cytoplasm and extracellular media after 45 min of experiment and to estimate the degree of maximal fluorescence (Supporting Information, Figure S2). Images were analyzed using NIS‐Elements and ImageJ software. Specifically, at least 10 ROIs per field were marked in mitochondrial (Rhod‐2) and cytoplasmic areas (Fuor‐4) in all the images, and a time measurement of the intensity in both fluorophores was conducted. The fluorescence for each time point was normalized to the fluorescence value measured at time = 0 s. and expressed as a percentage of this initial fluorescence. Results of typical experiment are shown in Supporting Information, Figure S2. Please note that under these experimental conditions, a spontaneous moderate increase on Fluo‐2 and Rhod‐2 fluorescence was observed, even under control conditions (Supporting Information, Figure S2a–b). Due to the time frame of these experiments, the peptide was pre‐aggregated as described above to obtain oligomers before being added to the cells.

### 4.10 TMRM assay

TMRM assay was performed using the probe in nonquenching mode conditions, as previously described in (Abramov, Fraley, et al., 2007). Cells were plated on 25‐mm optical borosilicate poly‐L‐lysine‐coated sterile glass covers (Thermo Fisher, Waltham, Massachusetts, USA) at a 70% confluence. The day after, cells were washed twice with HBSS and charged with 60 nM TMRM on HBSS. Cells were then incubated for 20 min in the incubator at 37°C and washed again with HBSS. After that, the medium was replaced by HBSS containing 15 nM of TMRM, to maintain the equilibrium distribution of the fluorophore. As shown in Supporting Information, Figure S1 under our experimental conditions, addition of FCCP leads to an abrupt drop in fluorescence. This confirms that TMRM fluorescence directly reflects the values of mitochondrial membrane potential and no appreciable quenching of TMRM fluorescence occurred. Cells were then mounted on Sykes‐Moore chambers (BellCo, Vineland, New Jersey, USA) and treated with the different CAIs and/or peptides. An image was taken at minute 0 and at minute 45, after adding the CAIs and/or peptides with different aggregation states.

### 4.11 pH measurement

Cells were plated on 96‐well fluorescence microtiter microplates (Thermo Fisher Scientific). The day after, cells were treated with the different CAIs and/or peptides. After treatment, intracellular pH measurement was performed using the pHrodo red AM kit (Thermo Fisher Scientific), following the indications provided by the manufacturer.

### 4.12 ATP measurement

Steady‐state levels of ATP were estimated using the Cell Titer‐Glo, according to manufacturer's instructions. In brief, 5 × 10^3^ cells per well of both ECs and SH‐SY5Y cells were plated on 96‐well plates. The day after, cells were treated with either 10 μM Q22 or 50 μM Ab42 peptides, respectively. Peptides were added alone or in combination with increasing concentrations of MTZ (100 and 300 μM) or ATZ (10 and 100 μM). Controls were treated with either DMSO or 5 μM oligomycin. After 3 hr of treatment, the plates containing the cells were equilibrated to room temperature for 30 min prior to addition of the luciferin/luciferase/cell lysis mixture. Absolute luminescence from quadruplicate experiments was recorded using a SpectraMax plate reader (Molecular Devices, Sunnyvale California, USA).

### 4.13 Statistical analysis

Statistical significance of differences between groups was determined by Student's *t* test or two‐tailed Student's *t* test. Moreover, in experiments containing more than two groups, the statistical significance was determined by ANOVA with turkey post‐hoc test. For the statistical analysis and the graphical representation, we used Origins Lab (Northampton, Massachusetts, USA) software and GraphPad Prism (GraphPad, La Jolla, CA). Values of *p* ≤ 0.05 were considered significant. (* *p* ≤ 0.05, **p ≤ 0.01, ***p ≤ 0.001).

## AUTHOR CONTRIBUTIONS

SF and MS wrote the manuscript and prepared figures. MS, PP, LD, and MSM performed experiments. SF supervised the study, designed research, edited the manuscript, and acquired funding. PP, MJDL, TW, and EP gave intellectual input and revised the manuscript.
