# Supplementary material for: Carbonic anhydrase inhibition selectively prevents amyloid β neurovascular mitochondrial toxicity
Source: Aging Cell. 2018 Jun 5;17(4):e12787. doi: 10.1111/acel.12787 (PMC6052473; doi:10.1111/acel.12787)
Supplement: Supplementary file 1 [file ACEL-17-na-s001.docx]

**SUPPORTING INFORMATION**

**
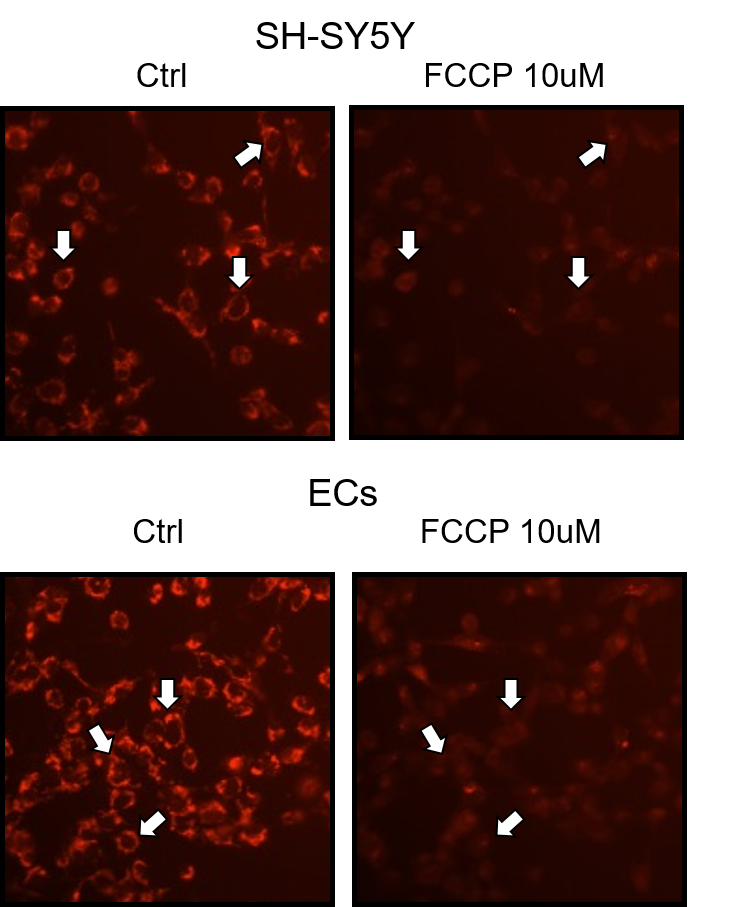
**

**Supporting Information, Figure 1.** Data showing mitochondrial membrane depolarization after adding FCCP 10uM to SH-SY5Y and ECs cells. Arrows mark individual mitochondria.


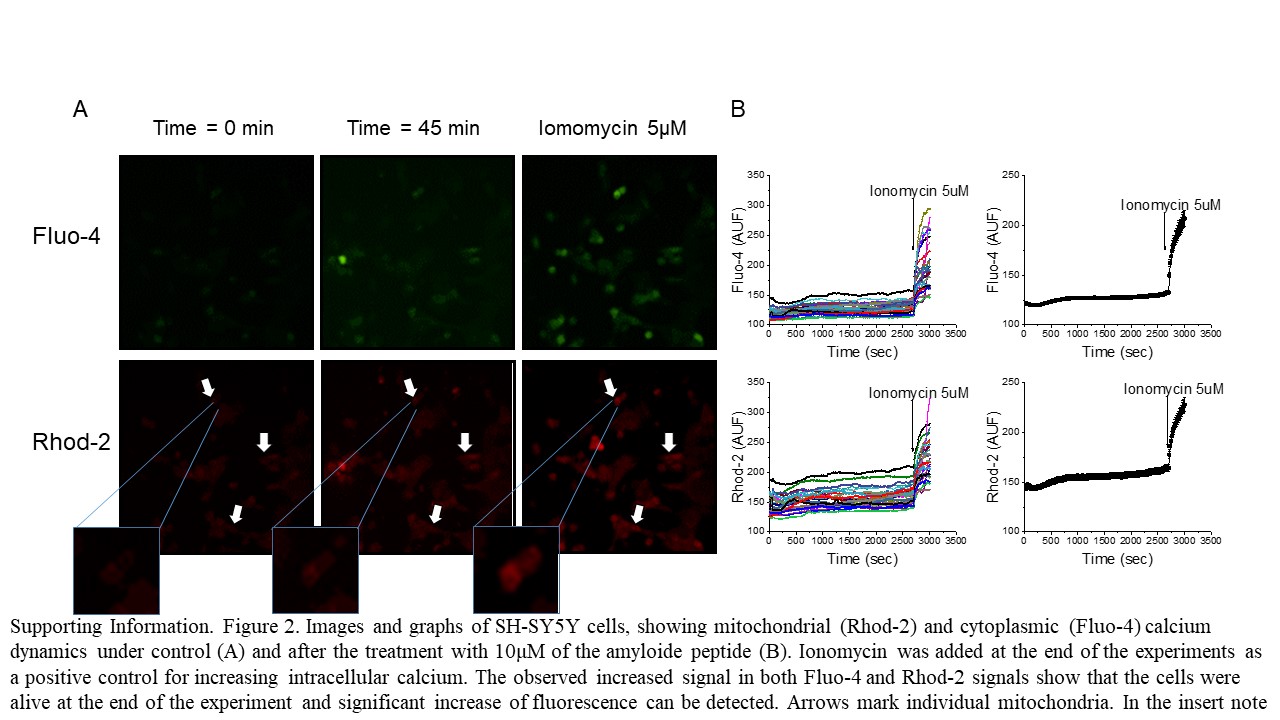


*
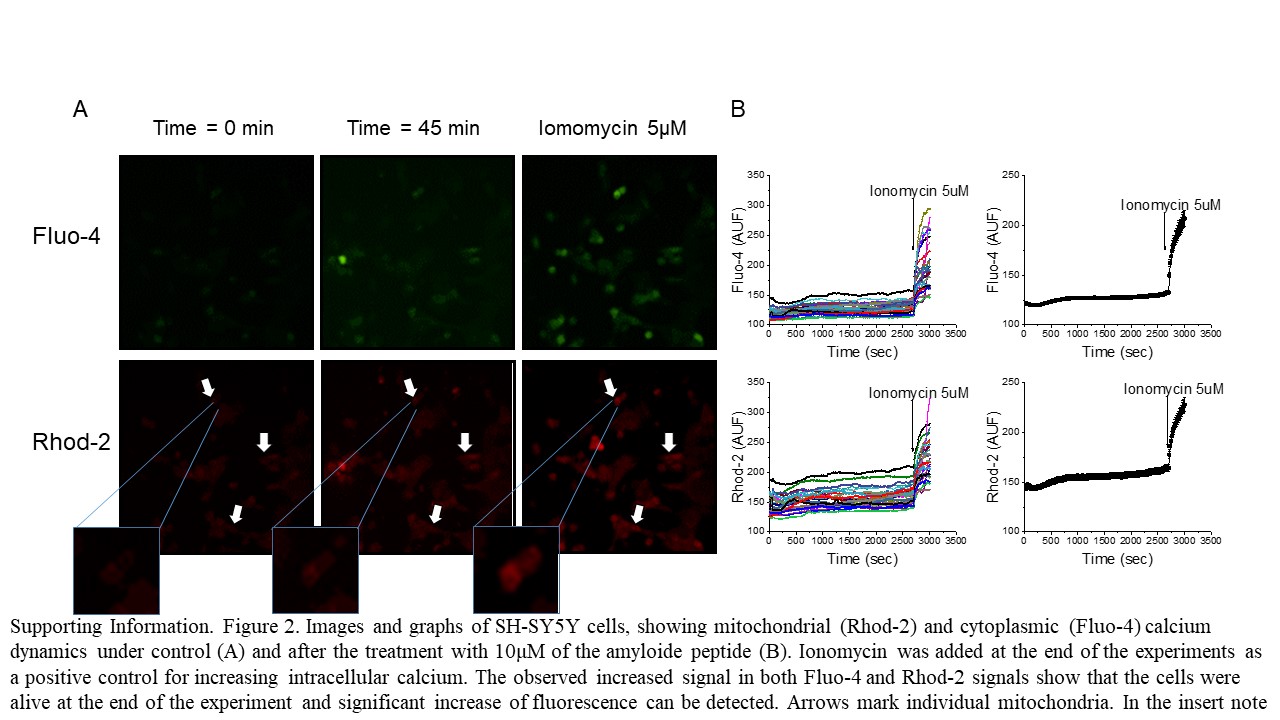
*

***Supporting Information, Figure 2.*** *(A) Images of SH-SY5Y cells, showing mitochondrial (Rhod-2) and cytoplasmic (Fluo-4) calcium fluorescence under control conditions (0 and 45 min.) and after ionomycin administration. Arrows mark individual mitochondria. Rhodamine-based probes show a preferential mitochondrial location, as previously reported (Smithen et al., 2013). (B) The corresponding graphs of 30 individual cells (left panels) and average signal (right panels). Ionomycin was added at the end of the experiments as a positive control for increasing intracellular calcium. The observed increase in both Fluo-4 and Rhod-2 signal shows that the cells were alive at the end of the experiment and significant increase of fluorescence can be detected after ionomycin addition.*
